# The effects of different doses of compound enzyme preparations on the production performance, meat quality and rumen microorganisms of yak were studied by metagenomics and transcriptomics

**DOI:** 10.3389/fmicb.2024.1491551

**Published:** 2024-12-11

**Authors:** Chenyang Zhang, YongFu La, Xiaoming Ma, Pingcuo Zhandui, Xiaoyun Wu, Xian Guo, Ping Yan, Luosan Dunzhu, Chunnian Liang

**Affiliations:** ^1^Key Laboratory of Yak Breeding Engineering of Gansu Province, Lanzhou Institute of Husbandry and Pharmaceutical Sciences of Chinese Academy of Agricultural Sciences, Lanzhou, China; ^2^Key Laboratory of Animal Genetics and Breeding on Tibetan Plateau, Ministry of Agriculture and Rural Affairs, Lanzhou, China; ^3^Institute of Animal Husbandry and Veterinary Medicine, Tibet Academy of Agriculture and Animal Husbandry Sciences, Lasa, China; ^4^Plateau Agricultural Science and Technology Innovation Center, Lasa, China

**Keywords:** compound enzyme, rumen microbial, performance, meat quality, metagenome, transcriptomics, yak

## Abstract

Yak (*Bos grunniens*) is a large ruminant endemic to the Tibetan plateau. The addition of enzyme complexes to feed can significantly improve their growth performance. Therefore, studying the effects of ruminant compound enzyme preparations dosage on yak rumen microorganisms and production performance is crucial to promoting the development of the yak industry. This study aimed to determine the effects of feeding yaks with different doses of ruminant enzyme compounds on the performance, meat quality, and rumen microorganisms of yaks. Three kinds of experimental diets with doses of 0.5 g/kg (LE group), 1 g/kg (ME group), and 2 g/kg (HE group) were selected to determine the growth index, meat quality, serum biochemical indexes, rumen fluid pH and other indexes of the three experimental groups. Metagenomics studies were used to investigate the differences in rumen microbial composition and function among yak groups, and transcriptome sequencing of the longest dorsal muscle was performed to reveal the expression of differential genes among different groups. It was determined that the levels of dietary enzyme complexes significantly affected growth performance, rumen fluid pH, and serum biochemical indices. At the phylum level, the dominant phylum in all three treatment groups was Bacteroidota, Bacillota, Kiritimatiellota, and Pseudomonadota. At the genus level, Prevotella, Methanobrevibacter, Oscillibacter. Fibrobacter showed statistically significant differences in abundance (*p* < 0.05). CAZymes family analysis revealed significant differences in GHs, CTs, and CEs among the three groups. Genome-wide differential gene expression in the longest muscle of the yak back was analyzed by RNA-seq between the three experimental groups. Some DEGs were found to be enriched in the ECM, PI3K-Akt, PPAR, and protein digestion and absorption receptor pathways. Combined metagenomics and transcriptomics analyses revealed that some microorganisms were significantly associated with the genes COL11A1, POSTN, and PTHLH, which are involved in growth metabolism. In summary, this study investigated the effects and interrelationships of ruminant complex enzymes on yak performance, meat quality, and rumen environment. The results of this study provide a scientific basis for adding ruminant enzymes to yaks.

## 1 Introduction

Yak is one of the important livestock and poultry resources in the plateau region. There are more than 17 million yaks in the world, and China has the largest number and breed group of yaks, accounting for 95% of the total number of yaks in the world. In China, yaks are mainly distributed in Tibet, Qinghai, Sichuan, and Gansu provinces (regions), and a few are distributed in neighboring Yunnan and Xinjiang (Fan et al., 2020). Yaks also provide many important products for people in the plateau area, namely milk, meat, fur and feces, as well as social status (Shah et al., 2023). They live in alpine environments such as the Qinghai-Tibet Plateau, with sparse vegetation, short plant growth cycles, low nutrients, and extreme climatic conditions (Ping et al., 2019). As a result, yak diets often contain large amounts of fiber and hard substances, such as rice straw, alpine grasses, and leaves, which are usually more difficult to digest and utilize. To help yaks digest their main food sources more efficiently, breeders commonly use enzyme complexes. Enzymes are active substance feed additives with high efficiency, specificity, and non-toxicity produced by modern biotechnological means through the fermentation of microorganisms such as fungi, bacteria, and actinomycetes in nature to produce proteins composed of amino acids Compound Enzyme (Dingyuan, 2019). Unlike single enzyme preparations, compound enzyme preparations can degrade multiple anti-nutritional factors and multiple nutrients at the same time and act synergistically with each other. This combination can help yaks break down complex polysaccharides and protein structures in their food, making them easier to digest and absorb (Waititu et al., 2018; Long et al., 2020; Srikanthithasan et al., 2020).

And when it comes to ruminants, we inevitably have to mention one of their unique physiological features—the rumen. The rumen hosts a large number of microorganisms, mainly including bacteria, fungi, and protozoa (Qiu et al., 2020). These microorganisms are capable of transforming the rumen into a healthy and productive environment through synergistic effects. These microorganisms are capable of converting plant fiber into volatile fatty acids (VFA) and microbial proteins (MCP) for their own and host utilization through synergistic interactions (Guo et al., 2020). The VFA produced can also provide a source of energy to the host and the plant. The VFA produced can provide 70–80% of the host’s energy requirements. Together with ruminants, these microorganisms build a complex microbial community that plays an important role in digestion and nutrient absorption in ruminants (Li et al., 2017). However, the function and structure of rumen microorganisms are affected by a variety of factors, such as diet, age, feeding management practices, genetics and the addition of exogenous microecological agent additives can affect the structure of rumen microorganisms in ruminants (Hongbo, 2015; Jiao et al., 2015; Yáñez-Ruiz et al., 2015; Simon et al., 2017; Li F. et al., 2019; Lintao, 2022). The rumen bacterial composition was investigated using 16S rRNA, and it was found that the addition of enzyme complexes to the diet significantly altered the relative abundance of rumen-dominant bacteria (Chunyan, 2010). The addition of enzyme complexes to diets not only affects the composition of microorganisms in the rumen but also influences host growth performance and serum biochemical indices (Zhi, 2023).

Although the effects of adding complex enzyme preparations on rumen microorganisms in ruminants have been extensively studied, most of the studies have been limited to the level of rumen bacteria using techniques such as 16S rRNA. Moreover, due to the differences in digestive and metabolic characteristics and environmental adaptations between yaks and other cattle breeds on the Qinghai Plateau, the effects on the microbial community may also be different (Guo et al., 2021). As microbial research has intensified, macroeconomics has been used to explore the effects of enzyme complexes added to rations on host traits and rumen microbial communities. In contrast to 16S rRNA technology, metagenomics not only allows for the study of bacterial composition and diversity in host microbes but also allows for in-depth genetic and functional studies (Ma et al., 2024). However, metagenomics has not been used to study the effects of different doses of complex enzyme preparations on yaks in the Qinghai Plateau.

In addition, whereas the analysis of muscle tissue using the transcriptome allows the identification of more candidate genes, regulatory networks, and signaling pathways to understand the genes involved in muscle growth. In muscle transcriptome analysis, differentially expressed genes, including up-and down-regulated genes, are available, which helps to study differences in growth rates and growth mechanisms. In addition to the effects at the gene level, the muscle transcriptome can also reveal the molecular mechanisms of growth differences caused by different feed conversions. Through transcriptome sequencing, the molecular mechanisms of growth differences induced by feed conversion can be better understood, providing a reference for optimizing feeds and improving growth performance. Muscle transcriptome studies are crucial for exploring the key factors of muscle growth and development, which will help future research on the role of muscle growth.

Therefore, the present study was conducted to verify the effects of different doses of enzyme composites in diets on growth performance, meat quality, and rumen microbial diversity, and analyzed the data from metagenomics and transcriptomics. The effects of different doses of enzyme composites on growth performance, meat quality, and rumen microbial diversity of yaks in the Qinghai Plateau were investigated. The correlation between serum and growth performance indices and rumen-dominant flora was analyzed, and the relationship between microbiota and growth was explored. It provides more scientific guidance for the fattening of yaks on the Qinghai Plateau and helps herders obtain more economic benefits.

## 2 Materials and methods

The experiments involved in this experiment were performed by the Lanzhou Institute of Animal Husbandry and Pharmaceutical Sciences of the Chinese Academy of Agricultural Sciences (CAAS; approval number: 1610322020018). All sampling procedures were performed in strict accordance with the Chinese Guidelines for the Ethical Handling of Laboratory Animals.

### 2.1 Animals, diets, and experimental design

The experiment was conducted in Qinghai. The main components of the compound enzyme preparation used in this experiment include xylanase 12,000 U/g, cellulase 500 U/g, *β*-glucanase 5,000 U/g, β-mannanase 1,000 U/g, pectinase 1,200 U/g, neutral protease 5,000 U/g and mesophilic *α*-amylase 800 U/g. In this experiment, 30 selected 3-year-old male yaks in good condition and close to the same weight were selected. The 30 yaks were randomly divided into three treatment groups using R software (Version 4.1.2). The feeding management was as follows: feed was fed twice a day during the experimental period at 8:00 and 16:00, and the feed intake was calculated by collecting and weighing the remaining feed before the morning feeding on the next day. The feeding amount was based on the production requirements, and all the test yaks were fed and watered freely (the feeding amount was sufficient, and the number of leftovers in each feeding was about 5%). During the test period, other feeding management measures were carried out according to the feedlot management system, and the pens were disinfected and cleaned regularly. Then the treatments of the three groups were: test LE group (feeding base concentrate +0.5 g/kg compound enzyme), test ME group (feeding base concentrate +1 g/kg compound enzyme), and test HE group (feeding base concentrate +2 g/kg compound enzyme), with 10 yaks in each treatment group. The feed formulation was designed concerning the recommended nutritional values for beef cattle in the Chinese beef cattle feeding standard (NY/T 815–2004) for beef cattle weighing 250 kg with a daily weight gain of 1 kg. The composition and content of the diets are shown in Supplemental material S1.

The experiment was divided into two phases: pre-test (15 d) and main test (120 d). The purpose of the experiment was to acclimatize the experimental animals to the supplemental diet in advance. After the pre-test was finished and the main trial started, the body weight of yaks was measured regularly every month.

### 2.2 Sample collection

Before morning feeding on the last day of the experiment, 20 mL of blood was collected from yaks by fasting, and the blood sample was centrifuged at a speed of 3,500 r/min for 12 min. After centrifugation, the upper layer of serum was collected, and the serum was dispensed into 1.5 mL AP centrifuge tube, which were brought back to the laboratory and stored in a refrigerator at −20°C, and then used for the analysis of biochemical indexes (automatic biochemical analyzer XL640 and biological kits were used to detect related indicators). After blood collection, four yaks were selected in each group to collect rumen fluid by oral catheter method. The cattle were held still, the mouth of the cattle was opened with an opener, and a rumen fistula was slowly inserted into the rumen from the cattle’s mouth, and rumen fluid was extracted. Fifty milliliter of rumen fluid was discarded initially (to avoid the influence of saliva), and about 60 mL was extracted again, and the rumen fluid was filtered through 4 layers of sterilized gauze and collected in the freezing tube, and then put into liquid nitrogen for quick-freezing, and then brought back to the laboratory to store the samples in the refrigerator at −80°C for the subsequent metagenomics sequencing analysis.

### 2.3 Slaughtering indicators and meat quality measurements

At the end of the experiment, 3 yaks with close average weight were randomly selected from each experimental group for slaughter (fasting for 24 h), and the carcass weight and eye muscle area were recorded. Then, 1 kg of meat samples were collected from the cross section of the 12–13 intercostal longissimus dorsi muscle of 12 cattle for transcriptome sequencing, and the pH, drip loss rate, cooking loss rate, shear force and water holding capacity of meat slices were determined according to the industry standard NY/T1180-2006 of the Ministry of Agriculture; the meat samples were placed in air to be oxidized for 45 min, and the color of the meat was determined by a colorimeter, and the average was taken as the value of the color of the meat.

### 2.4 DNA extraction, metagenomics sequencing, data processing

Total microbial DNA was extracted from 12 rumen fluid samples using a DNA extraction kit [QIAamp^®^FastDNAStoolMiniKit (Qiagen, Hilden, Germany)]. NanoDrop2000 spectrophotometer (ThermoFisherScientific, Waltham, MA, United States) and agarose gel electrophoresis for DNA concentration determination and integrity analysis. DNA fragmentation was performed using S220Focused-ultrasonicators (Covaris, United States) and purification was performed by AgencourtAMPureXPbeads (BeckmanCoulterCo., United States). TruSeqNanoDNA LTSamplePrepararionKit (Illumina, SanDiego, CA, United States) kit was used for library construction.

The libraries were sequenced using the lluminaNovaseq6000 sequencing platform and 150 bp double-ended reads were generated. The raw downstream data (FASTQC) was disjointed using Fastp (v0.20.1) and filtered to remove low-quality bases, and reads containing N bases (fuzzy bases) were removed (Chen et al., 2018). Using BBMap (v38.93-0) to compare with the host genome and remove host sequences (Bushnell, 2014). After obtaining valid reads, metagenomics splicing was performed using MEGAHIT (v1.2.9) (Dinghua et al., 2015; Li et al., 2016). ORF prediction of spliced scaffolds was performed using prodigal (v2.6.3) and translated into amino acid sequences (Hyatt et al., 2010). Non-redundant gene sets were constructed using MMSeqs2 (v13.45111) for the predicted genes in all the samples, with clustering parameters of 95% identity and 90% coverage between sequences. The longest gene in each cluster was selected as the representative sequence of the gene set, and after obtaining the representative sequences of the gene set, the gene set representative sequences of each sample were analyzed using salmon (v1.8.0) to compare the clean reads of each sample with the non-redundant gene set separately (95% identity), and statistically the abundance information of the genes in the corresponding sample.

Species annotations were obtained from the taxonomic information database corresponding to the NR library, and then the abundance of the species was calculated using a combination of the corresponding gene abundances of the species. The abundance of species in individual samples was counted at the Phylum and Genus taxonomic levels to construct abundance profiles at the corresponding taxonomic levels. The representative sequences (amino acid sequences) of the gene sets were annotated against the NR library, KEGG, GO, and so on using the DIAMOND (v0.9.10.111) software, and the BLAST comparison parameters were set to have an expected *e*-value of 1e-5 (Huson and Buchfink, 2015). The gene set was compared with the CAZy database using the CAZy database correspondence tool, hmmscan (v3.1), to obtain the annotation information of the carbohydrate-active enzymes corresponding to the genes, and then the abundance of the carbohydrate-active enzymes was calculated using the sum of the abundance of the genes corresponding to the carbohydrate-active enzymes.

The vegan in the R package is used to perform PCA analysis and mapping on the species abundance spectrum or functional abundance spectrum, to calculate the results of PCoA, NMDS, and other distance matrices, and to perform graphical analysis. Based on vegan in R package, Kruskal Wallis was used for variance analysis. LEfSe was used for variance analysis of species abundance spectra or functional abundance spectra.

### 2.5 RNA extraction, transcriptome sequencing, data processing

Total RNA was extracted from 12 muscle samples using TRIzol reagent according to the instructions, RNA purity and quantification were determined using a NanoDrop 2000 spectrophotometer (Thermo Scientific, United States), and RNA integrity was assessed using an Agilent 2100 Bioanalyzer (Agilent Technologies, Santa Clara, CA, United States) to assess RNA integrity. Transcriptome libraries were constructed using the VAHTS Universal V5 RNA-seq Library Prep kit according to the instructions.

The libraries were sequenced using the Illumina Novaseq 6000 sequencing platform, and 150 bp double-ended reads were generated. Fastp software was used to process the raw reads in fastq format, and clean reads were obtained after removing the low-quality reads for subsequent data analysis. HISAT2 software was used for reference genome comparison and FPKM calculation, and HTSeq-count was used to obtain the counts of reads for each gene. Using ggplot2 in the R package to perform PCA analysis and mapping of counts to evaluate sample biological replicates. Differentially expressed genes were analyzed using DESeq2 software, where genes that met the thresholds of *q*-value < 0.05 and foldchange >2 or foldchange <0.5 were defined as differentially expressed genes (DEGs). Hierarchical clustering analysis of DEGs was performed using R (v 3.2.0) to demonstrate the expression patterns of genes across groups and samples. The ggradar in the R package was used to draw radar maps of genes to show changes in the expression of up-regulated or down-regulated genes, GO, KEGG Pathway enrichment analysis of differentially expressed genes based on the hypergeometric distribution algorithm was used to screen for significantly enriched functional entries. Bar graphs were plotted against significantly enriched functional entries using ggplot2 in the R package. Gene set enrichment analysis was performed using GSEA software. Using a predefined set of genes, genes were sorted according to the degree of differential expression in the two sample types and then tested whether the predefined set of genes was enriched at the top or bottom of this sorted list.

### 2.6 Correlation analysis between metagenomics and transcriptome

The correlation analysis shows the correlation between the characteristics of different groups. The metagenomics and transcriptome data were screened according to the protogenomic difference criteria, and then the correlation coefficients between all the differences were calculated. This analysis uses spearman correlation algorithm. The top 30 items with significant differences between metagenomics and transcriptome were selected at the phylum level and the genus level (based on *p*-value ranking, all significant difference results were used when less than 30), and the correlation between them was calculated. The correlation matrix heat map was drawn by ggplot2 in the R package.

### 2.7 Statistical analysis

One-way analysis of variance (ANOVA) was used to analyze the monthly weight data and weight changes of yaks in each group of randomized block design using SPSS (Version 26). Statistical results were expressed as mean and standard deviation. They were then visualized using GraphPad (Version 9.0). Blood biochemical indices and growth performance as well as meat quality were tested for normality and chi-square using SPSS (Version 26) software, and differences among the three groups were analyzed by ANOVA, with *p* < 0.05 being considered statistically significant. Finally, the results were expressed as the standard deviation (SD) of the means.

## 3 Results

### 3.1 Changes in growth indices

**Figure 1:**
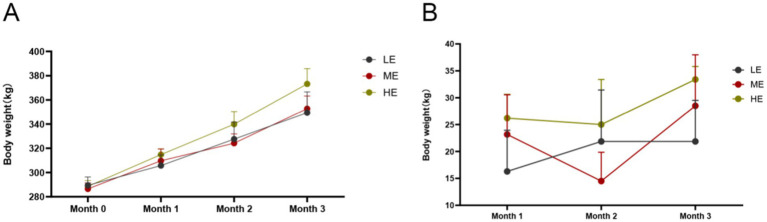
Effects of different dosages of complex enzymes on body weight **(A)** and body weight change **(B)**. It is represented by the mean and standard deviation (SD) of each group. LE, low dose group; ME, medium dose group; HP, high dose group (LE is 0.5 g/kg for low dose group; ME is 1 g/kg for medium dose group: HE is 2 g/kg for high dose group).

The effects of different doses of ruminant complex enzymes on body weight and monthly weight changes of yaks during the positive test period are shown in Figures 1A,B. According to SPSS, ANOVA was conducted to analyze the body weights and body weight changes of the three groups (Supplementary material S2), in the first month, the difference in monthly average body weights of the three groups was not significant, but the second month the monthly average body weight of the HE group was significantly higher than that of the LE group (*p* < 0.05), and in the third month the HE group was higher than that of the ME group and LE group (*p* < 0.05). In terms of monthly weight gain as seen in Figure 1B, yaks in LE, ME, and HP groups were in a state of weight gain until the end of the formal trial. In terms of daily weight gain, HE group > ME group > LE group. The HE group was significantly higher than the ME and LE groups (*p* < 0.05).

### 3.2 Changes in serum biochemical indices and rumen fluid pH

**Figure 2:**
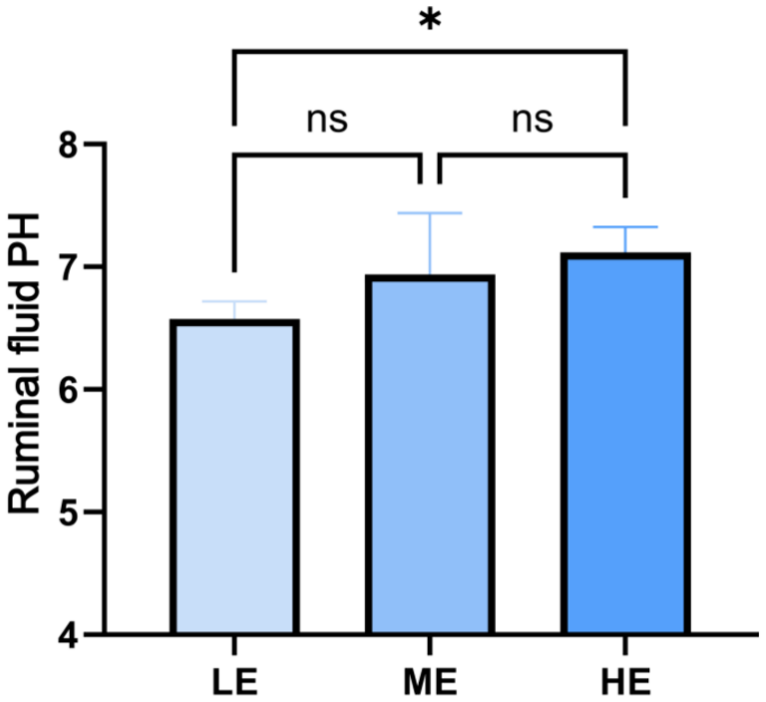
Effects of different doses of compound enzyme on rumen fluid pH. The mean and standard deviation (SD) of each group were expressed. “*” means correlation *p*-value < 0.05 (LE is 0.5 g/kg for low dose group; ME is 1 g/kg for medium dose group: HE is 2 g/kg for high dose group).

The effects of the three treatments on the serum biochemical indexes of yaks are shown in Table 1, and the changes of rumen fluid pH are shown in Figure 2. In terms of serum biochemical indexes, only the calcium and blood glucose concentrations of the LE and ME groups were significantly higher than those of the HE group (*p* < 0.05), and there were no significant differences in the other indexes among the three groups. In terms of rumen fluid pH, the HE group > ME group > LE group, and the rumen fluid pH of the HE group was significantly higher than that of the LE group (*p* < 0.05).

**Table 1:** Three treatment groups of yak serum biochemical indexes.

Item	Treatment group (mean ± SEM)			*p*-value
	LE	ME	HE	
TP (total protein g/L)	78.05 ± 9.49	78.48 ± 6.18	79.86 ± 4.45	0.865
ALB (albumin g/L)	25.61 ± 2.6	26 ± 2.06	26.25 ± 2.31	0.866
GLO (globulin g/L)	51.01 ± 6.99	52.48 ± 4.66	54.12 ± 3.61	0.519
A/G (albumin/globulin)	0.51 ± 0.06	0.51 ± 0.033	0.47 ± 0.071	0.182
AST (glutamic-oxalacetic transaminase U/L)	81.29 ± 9.5	77 ± 9.36	76.29 ± 14.22	0.682
ALP (alkaline phosphatase U/L)	128.67 ± 40.83	137.67 ± 24.47	132.33 ± 54.96	0.901
LDH (lactic dehydrogenase U/L)	998 ± 175.5	928.33 ± 166.39	974.89 ± 181.52	0.693
CREA (creatinine mmol/L)	189.03 ± 50.34	153.56 ± 65.32	171.88 ± 47.17	0.404
GLU (blood glucose concentration mmol/L)	8.96 ± 0.42^a^	8.99 ± 0.54^a^	7.96 ± 0.71^b^	0.004
CA (calcium mmol/L)	3.87 ± 0.11^a^	3.86 ± 0.14^a^	3.71 ± 0.12^b^	0.024
TG (triglyceride mmol/L)	0.25 ± 0.05	0.23 ± 0.066	0.26 ± 0.075	0.649
HDL (high density lipoprotein mmol/L)	1.47 ± 0.55	1.47 ± 0.44	1.28 ± 0.54	0.681
LDL (low density lipoprotein mmol/L)	0.21 ± 0.11	0.18 ± 0.081	0.14 ± 0.078	0.298
TCH (total cholesterol mmol/L)	1.91 ± 0.74	1.89 ± 0.63	1.53 ± 0.7	0.438
TBIL (total bilirubin mg/dL)	2.37 ± 1.09	1.59 ± 0.56	1.81 ± 0.37	0.091
ALT (glutamic pyruvic transaminase U/L)	27.6 ± 5.12	26.25 ± 5.59	26.14 ± 4.91	0.823

The values of each treatment group were expressed as mean ± standard error. In the same row, the superscripts of different lowercase letters indicate significant differences between groups (LE is 0.5 g/kg for low dose group; ME is 1 g/kg for medium dose group: HE is 2 g/kg for high dose group).

### 3.3 Meat quality measurement results

According to SPSS, the analysis of variance (ANOVA) of the meat quality measurement data of the three groups is shown in Table 2. The results showed that the differences among the three groups were not significant for meat color 45 min brightness L, meat color 45 min redness a, meat color 45 min yellowness b, meat color 24 h brightness L, meat color 24 h redness a, meat color 24 h yellowness b, pH45min, pH24h, pH48h, drip loss rate 24 h, drip loss rate 48 h, cooking loss rate, shear, carcass weight, and eye muscle area content were not significantly different.

**Table 2:** Meat quality measurement data of three experimental groups.

Item	Treatment group (mean ± SEM)			*p*-value
	LE	ME	HE	
Meat color 45 min brightness L	11.52 ± 2.14	10.7 ± 0.72	11.11 ± 1.29	0.752
Meat color 45 min redness a	23.77 ± 3.43	21.7 ± 1.79	23.44 ± 2.63	0.531
Meat color 45 min yellow b	4.28 ± 0.8	3.85 ± 0.37	4.42 ± 0.84	0.518
Meat color 24 h brightness L	10 ± 0.99	10.62 ± 0.8	11.02 ± 1.87	0.561
Meat color 24 h redness a	21.59 ± 1.27	22.85 ± 1.39	23.44 ± 2.74	0.417
Meat color 24 h yellow b	3.97 ± 0.73	4.54 ± 0.59	4.59 ± 1.08	0.526
PH45 min	6.27 ± 0.52	6.14 ± 0.58	5.81 ± 0.65	0.541
PH24 h	6.06 ± 0.52	5.87 ± 0.93	5.89 ± 0.17	0.894
PH48 h	6.59 ± 0.71	6.54 ± 0.65	6.38 ± 0.35	0.870
Drip loss rate of 24 h	0.038 ± 0.014	0.039 ± 0.01	0.04 ± 0.012	0.959
Drip loss rate 48 h	0.053 ± 0.015	0.055 ± 0.016	0.049 ± 0.013	0.859
Cooking loss rate	0.358 ± 0.188	0.28 ± 0.047	0.276 ± 0.48	0.154
shear force (unit: kgf)	11.23 ± 3.38	12.01 ± 0.74	10.72 ± 4.55	0.927
Carcass weight (unit: kg)	390.8 ± 41.36	403.2 ± 44.89	468.9 ± 69.55	0.175
eye muscle area (unit: cm^2^)	74.2 ± 7.02	76.06 ± 16.88	71.96 ± 11.16	0.913

The values of each treatment group were expressed as mean ± standard error. In the same row, the superscripts of different lowercase letters indicate significant differences between groups (LE is 0.5 g/kg for low dose group; ME is 1 g/kg for medium dose group: HE is 2 g/kg for high dose group).

### 3.4 Metagenomics data statistics

A total of 911,585,188 raw reads were obtained from the 12 samples in the three treatment groups, with an average of 75,965,432 ± 4,698,036 raw reads per sample. After quality control, a total of 909,579,992 clean reads were retained, with an average of 75,798,332.67 ± 4,664,460 clean reads per sample (Supplementary material S3). *Ab initio* assembly of the retained data generated a total of 17,770,215 contigs with an average of 1,480,851.25 ± 238,493.08 contigs per sample, of which the length of the N50 per sample was 808.92 ± 66.95 bp. gene prediction showed that all samples had 20,162,770.00 open reading frame orf. 94.1% for bacteria, 1.05% for eukaryotes, 4.04% for archaea, and 0.81% for viruses (Supplementary material S4).

### 3.5 Microbial profiles of the three treatment groups

**Figure 3:**
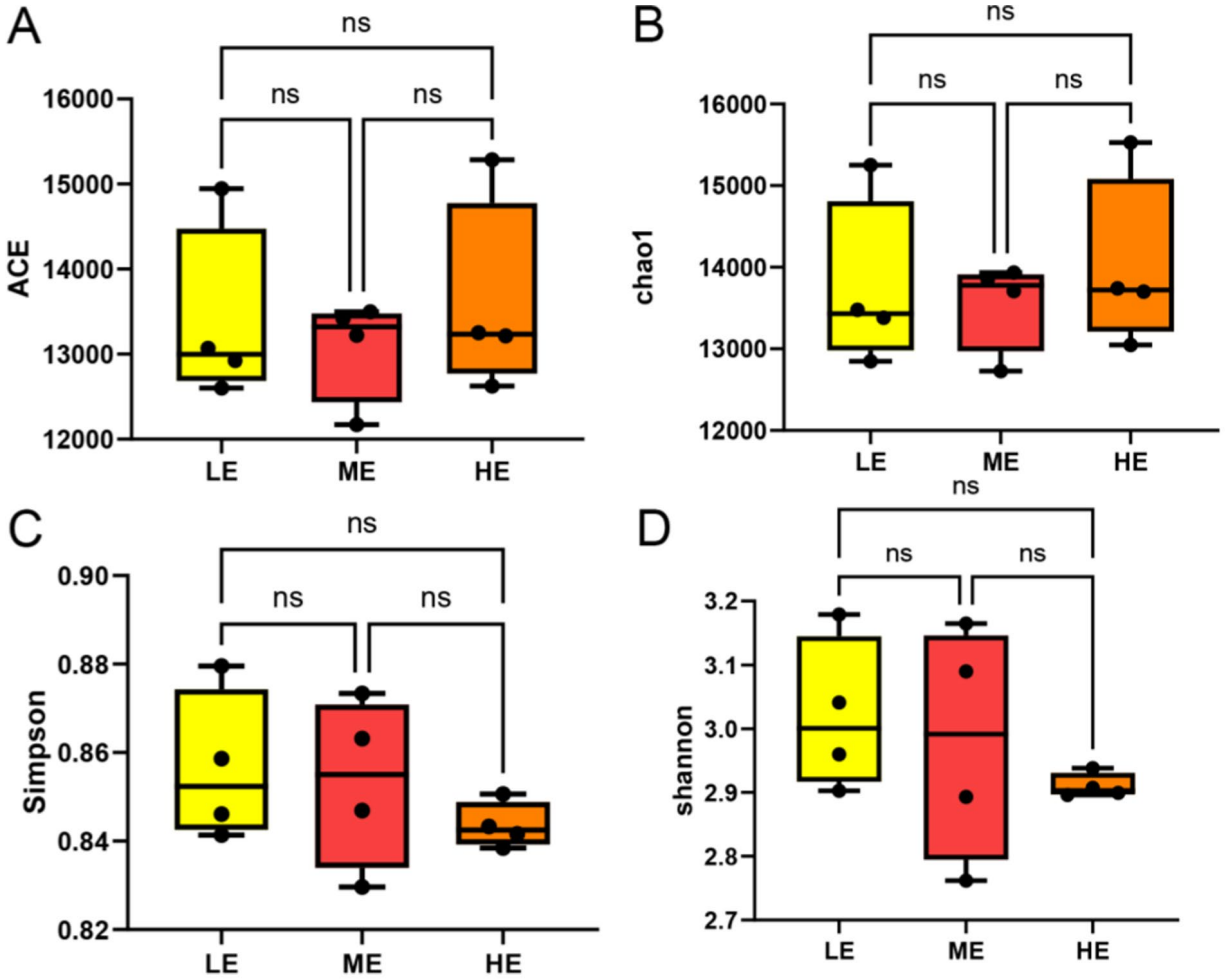
The ruminal microbial community *α* diversity index between different doses of compound enzyme preparations. ACE **(A)**, Chao1 **(B)**, Simpson **(C)**, Shannon **(D)**. The mean and standard deviation (SD) of each group were expressed (LE is 0.5 g/kg for low dose group; ME is 1 g/kg for medium dose group: HE is 2 g/kg for high dose group).

**Figure 4:**
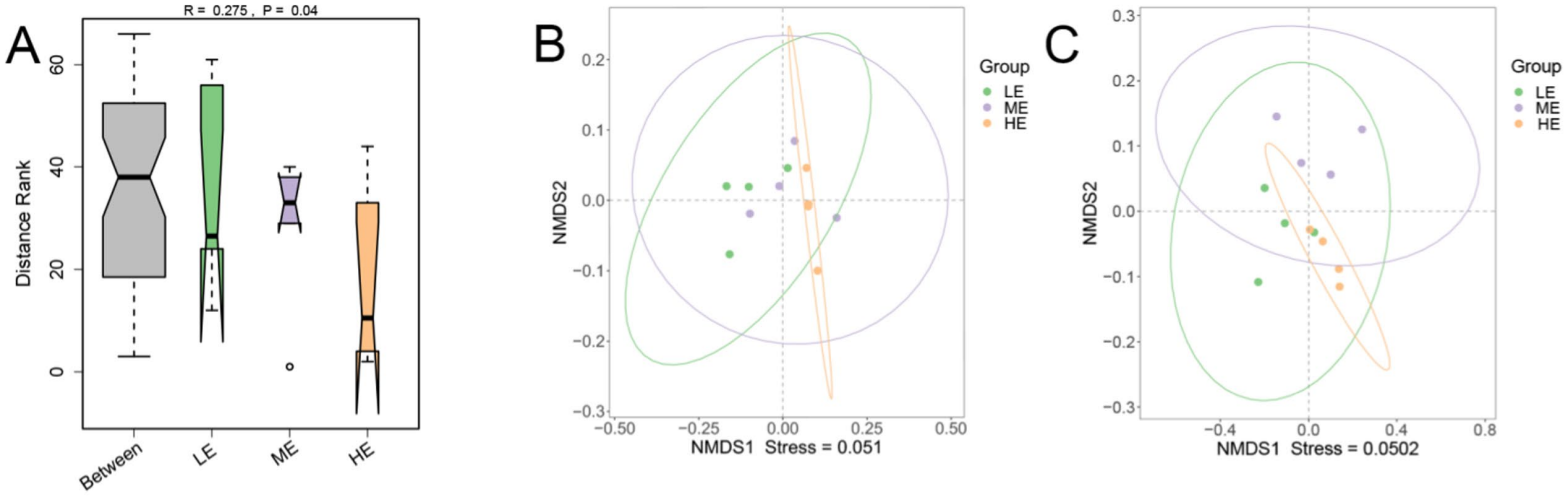
Anosim analysis **(A)** at the genus level and non-metric multidimensional analysis (NMDS) of microbial composition at the phylum **(B)** and genus **(C)** levels were performed in the three treatment groups (LE is 0.5 g/kg for low dose group; ME is 1 g/kg for medium dose group: HE is 2 g/kg for high dose group).

**Figure 5:**
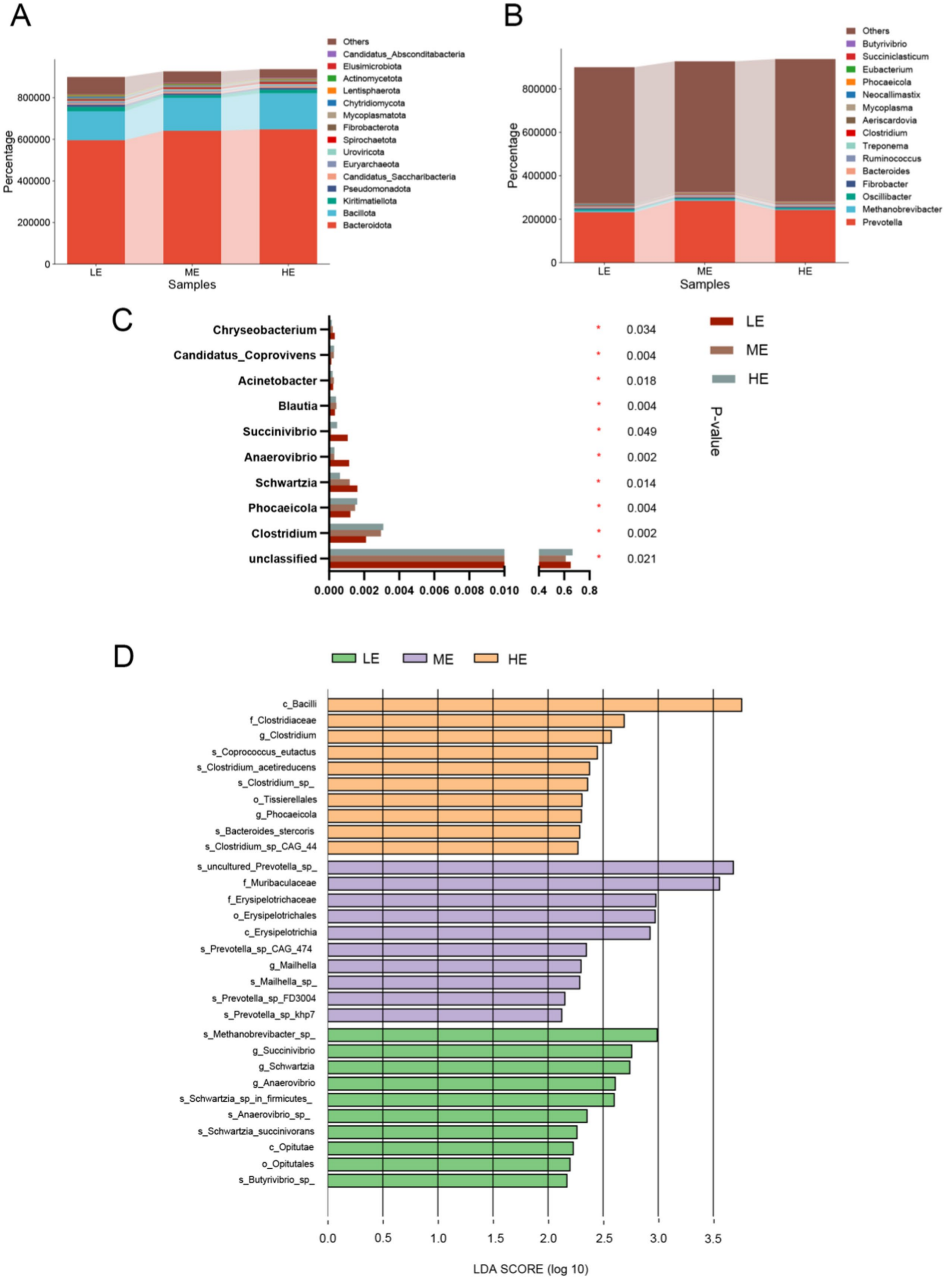
Microbial community composition and differential analysis of the three treatment groups. Microbial community abundance at phylum **(A)** and genus **(B)** levels for the three treatment groups. **(C)** Kruskal-Wallis H test to compare differences in microbial composition of the three treatment groups at the genus level. “*” means correlation *p*-value < 0.05. **(D)** The most representative biomarkers in each treatment group were identified at the genus level using the LDA Effect Size (LEfSe) algorithm. *Indicates that Microbials are significantly different among groups (LE is 0.5 g/kg for low dose group; ME is 1 g/kg for medium dose group: HE is 2 g/kg for high dose group).

Comparison of the differences in rumen microbial community *α*-diversity indices among the three groups of yaks, LE, ME, and HE, revealed that the ACE index and Chao1 of the rumen microbial community were the highest in the HE group (Figures 3A,B), and Simpson and Shannon were the highest in the LE group (Figures 3C,D), but the differences among the three groups were not significant (*p* > 0.05). The results of Anosim analysis of β-diversity showed that the difference between groups was greater than that within groups, indicating that the difference between the two groups was large (Figure 4A). The microbial composition of 12 samples (phylum and genus level) was analyzed by non-metric multidimensional scale analysis (NMDS) and analyzed and plotted using the vegan package of R software. The results showed that the three treatment groups had good clustering (Figures 4B,C) for subsequent studies. A total of 204 phylum, 3,910 genera, and 19,414 species were identified in all samples by comparison with the Nr database. Visualization of microbial composition at phylum and genus levels based on the three treatment groups showed that at the phylum level, the dominant phyla in the three treatment groups were Bacteroidota, Bacillota, Kiritimatiellota, and Pseudomonadota, among others (Figure 5A). At the genus level, the dominant microbial genera in the three treatment groups mainly included Prevotella, Methanobrevibacter, Oscillibacter, Fibrobacter, etc. (Figure 5B). To investigate whether species differed significantly between groups, Kruskal-Wallis rank sum test was performed on microbial genus samples from the three treatment groups (Figure 5C). Schwartzia, Anaerovibrio, Succinivibrio, and Chryseobacterium were significantly higher in the LE group than in the ME and HE groups (*p* < 0.05). The levels of Phocaeicola, Clostridium, Blautia, and Candidatus_Coprovivens were significantly higher in the ME and HE groups than in the LE group (*p* < 0.05). A linear discriminant analysis of effect size (LDA) coupled with effect size measurements, with an LDA threshold of 2, was used to screen microorganisms that showed significant differences among the three treatment groups (Figure 5D). Differential microorganisms identified in the LEfSe analysis containing the flora observed in the rank sum test confirmed the differential microorganisms in the different groups.

### 3.6 CAZyme composition

**Figure 6:**
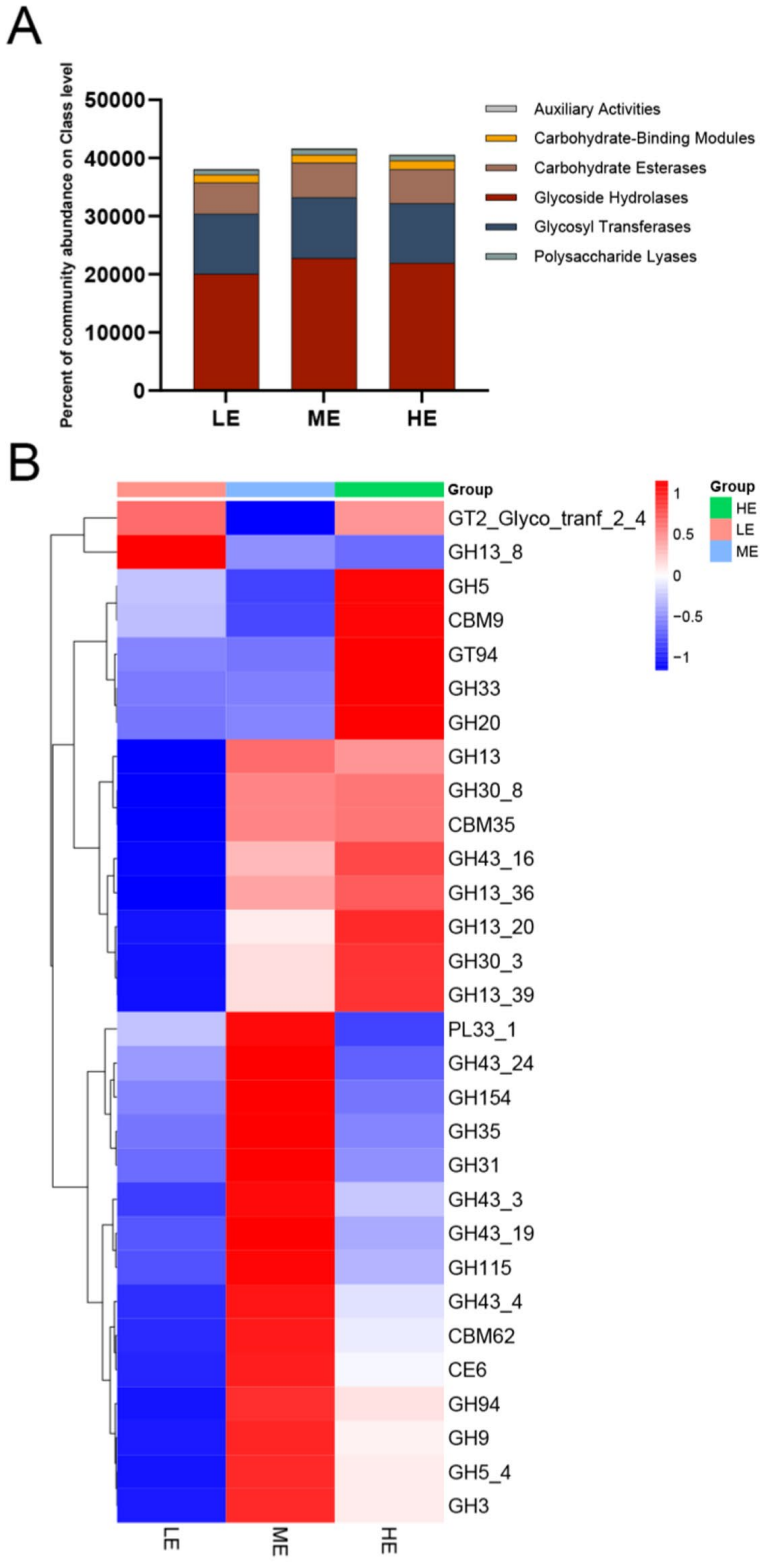
Metagenomic data from the three treatment groups were subjected to carbohydrate active enzyme analysis. **(A)** The proportion of CAZyme abundance at the class level in the three treatment groups. **(B)** The proportion of CAZyme abundance at family level in the three treatment groups (LE is 0.5 g/kg for low dose group; ME is 1 g/kg for medium dose group: HE is 2 g/kg for high dose group).

Ruminal microorganisms can produce a wide range of complex CAZymes, and obtaining annotated information on CAZyme genes is crucial for revealing the mechanism of microbial carbohydrate metabolism. A total of 432 CAZyme genes were identified in this study. There were 234 glycoside hydrolases (GHs), 72 glycosyltransferases (GTs), 53 polysaccharide lyases (PLs), 53 carbohydrate-binding modules (CBMs), 15 carbohydrate esterases (CEs), and 5 auxiliary active enzymes (AAs). Figure 6A shows the changes in the relative abundance of CAZymes responsible for decomposing diets with different energy levels in different groups.

Heatmap was plotted by differential function Heatmap plot, screening for significant differential function based on *p*-value, taking the information of CAZyme family with abundance TOP30. (Figure 6B), which revealed significant differences between the experimental groups fed different levels of complex enzyme preparations. GH5, CBM9, GT94, GH33, GH20, GH30_8, CBM35, GH43_16, GH13_36, GH13_20, GH30_3, and GH13_39 were found to be higher in the HE group. In the ME group, the levels of GH13, PL33_ 1, GH43_24, GH154, GH35, GH31, and 16 other CAZyme families were significantly higher in abundance.28 CAZyme families were lower in the LE group and only GT2_Glyco_tranf_2_4 and GH13_8 were higher in abundance, suggesting that feeding different doses of complex enzyme preparation affects the genes encoding carbohydrate-active enzymes.

### 3.7 Transcriptome sequencing quality analysis

Due to the contamination of the HE3 group samples of the transcriptome, 11 samples were sequenced, yielding a total of 52,656,000 raw reads. The average raw reads per sample were 47,870,000 ± 690,000 reads. After removing connectors, n-containing reads and low-quality reads, the average number of clean reads obtained in each group was 46.94 million ±630,000 reads, and the qualification rate was above 93.37%. The Q30 values ranged from 93.37% ~ 95.71%, which fulfilled the requirement of ≥90% of the Q30 sequences, and the GC content of the 11 samples ranged from 53.35% ~ 54.87%. The quality of the reads was good and could be further analyzed.

The clean reads of the samples were quickly and accurately localized to the reference yak genome (BosGru_v2.0; GCA_000298355.1) using HISAT2 software (v 2.0.5) with a localization rate of 91.34% ~ 93.62%. The comparison results are shown in (Supplementary material S5).

### 3.8 Gene expression level analysis

**Figure 7:**
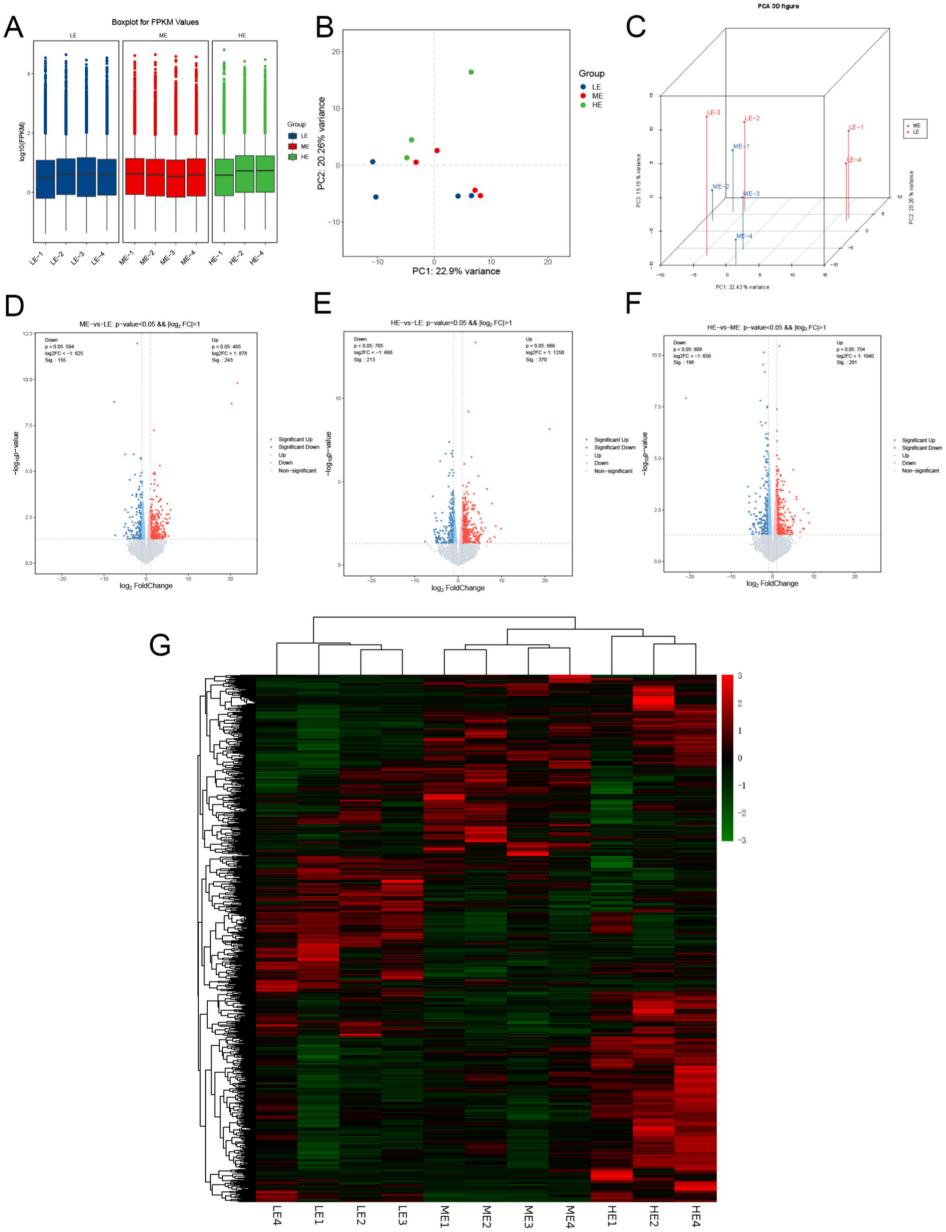
Analysis of differentially expressed genes in three treatment groups: **(A)** Box plot of gene expression level of samples. **(B)** Principal component analysis (PCA) diagram. **(C)** PCA 3D diagram of ME vs. LE. Volcano maps of the number of differentially expressed genes between **(D)** ME group and LE group, **(E)** HE group and LE group, **(F)** HE group and ME group. **(G)** Cluster analysis of the number of differentially expressed genes (LE is 0.5 g/kg for low dose group; ME is 1 g/kg for medium dose group: HE is 2 g/kg for high dose group).

Box-and-line plots (box-and-line plots) were used to describe the expression levels of the sample genes (Figure 7A), which showed low dispersion, good reproducibility, and good overall expression. Principal component analysis (PCA) plots showed (Figures 7B,C) that the samples were dispersed between groups and clustered within groups, indicating that the samples in this experiment were selected reasonably and the samples were reproducible.

### 3.9 Statistics and analysis of differentially expressed mRNAs

Based on the good reproducibility of the biological samples, DESeq2 software (v 1.16.1) was used to analyze the differential transcription between the two combinations. According to the differential screening condition (|log2FC| ≥ 0 and *p*-value ≤ 0.05), the LE and ME and HE groups were compared two by two, and 398, 583, and 498 differentially expressed genes were obtained, respectively. On the volcano plots (Figures 7D–F), the overall distribution of differential genes, as well as the distribution of differential genes in each comparison pair, is shown. All differential genes from the experimental groups were collected as differential gene sets; FPKM values were used for cluster analysis to better understand the gene expression patterns in different experimental groups. The results of the cluster analysis in the figure showed (Figure 7G) that differentially expressed genes in different experimental groups were grouped into one category for genes with up-regulated expression and one category for genes with down-regulated expression, respectively. Included in these DEGs are many genes that may be associated with the regulation of muscle, bone, and nervous system development, such as COL11A1, FOXO1, POSTN, and PTHLH.

### 3.10 GO and KEGG analysis of DEGs

**Figure 8:**
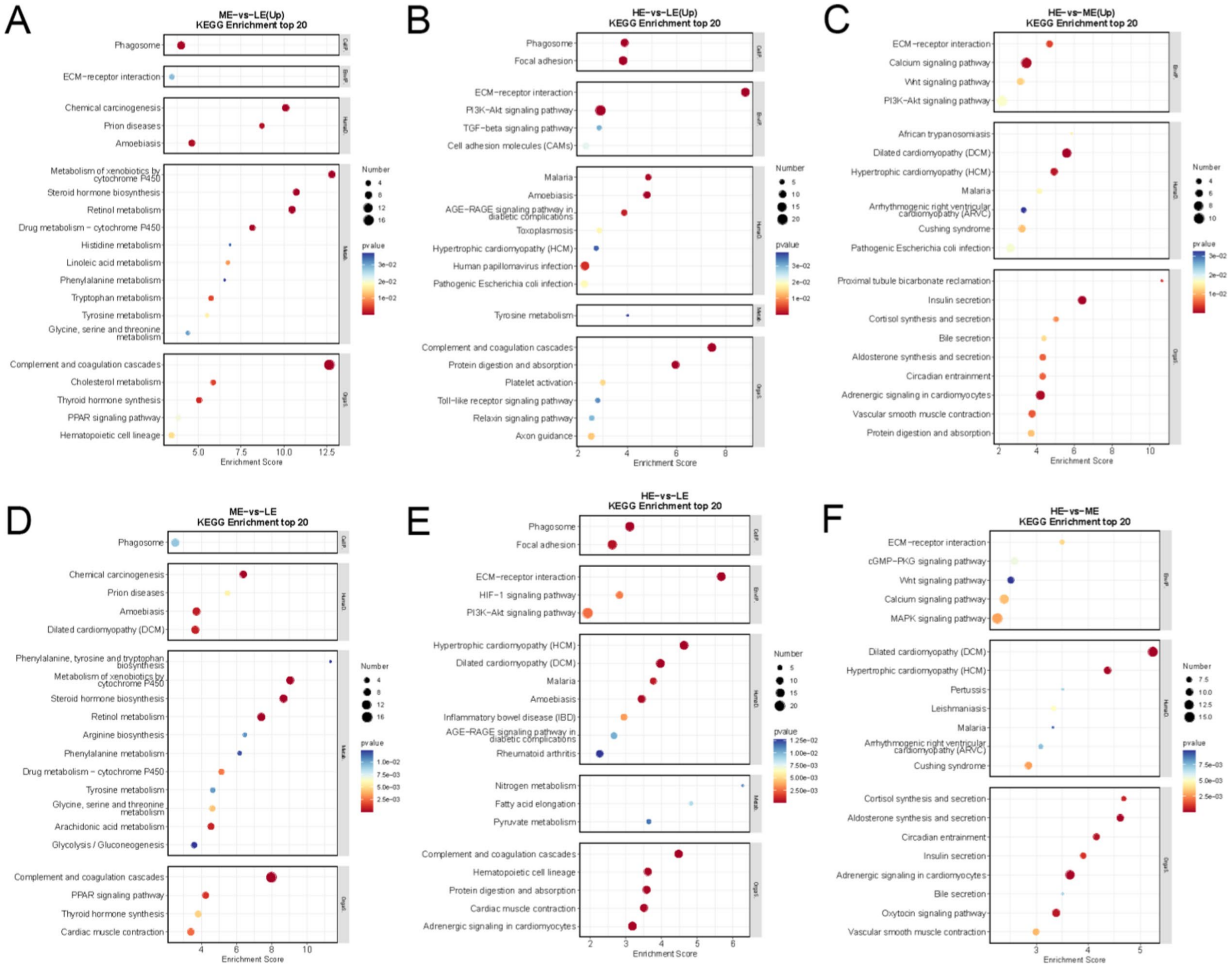
The scatter diagram of KEGG enrichment analysis of differentially expressed genes: **(A)** ME group and LE group, **(B)** HE group and LE group, **(C)** HE group and ME group top 20 significantly enriched KEGG pathways. **(D)** ME group and LE group, **(E)** HE group and LE group, **(F)** HE group and ME group, the first 20 significantly up-regulated KEGG pathways (LE is 0.5 g/kg for low dose group; ME is 1 g/kg for medium dose group: HE is 2 g/kg for high dose group).

Analysis of KEGG pathways showed that DEGs were significantly enriched in 34 pathways in the ME and LE groups. Compared with the LE group, production performance-related ECM-receptor interaction, PPAR signaling pathway, Glycine, serine, and threonine metabolism were significantly up-regulated in the ME group compared with the LE group (Figure 8A); DEGs were significantly enriched in 40 pathways in the HE and LE groups, among which ECM-receptor interaction, PI3K-Akt signaling pathway, Protein digestion, and absorption pathway were significantly up-regulated in the ME group compared with the LE group (Figure 8B); DEGs were significantly enriched in 47 pathways in the HE and ME groups, of which the PI3K-Akt signaling pathway, Insulin secretion, ECM-receptor interaction pathway was significantly upregulated (Figure 8C). The top 20 significantly enriched (*p* < 0.05) KEGG pathways were selected between the two groups for demonstration (Figures 8D–F).

**Figure 9:**
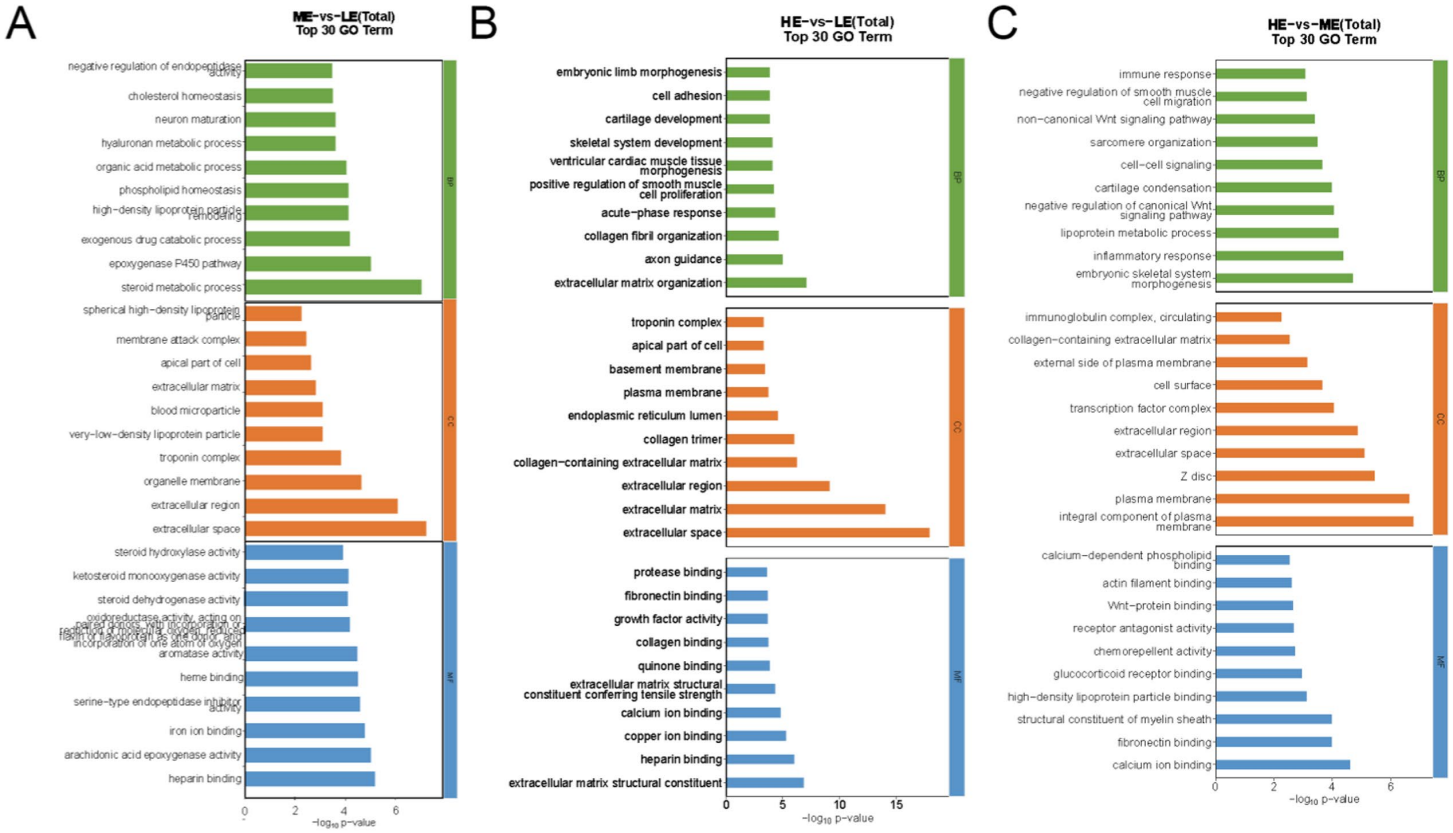
The bar chart of GO enrichment analysis of differentially expressed genes: **(A)** ME group and LE group, **(B)** HE group and LE group, **(C)** HE group and ME group The top 10 terms with the highest significance in each functional category (LE is 0.5 g/kg for low dose group; ME is 1 g/kg for medium dose group: HE is 2 g/kg for high dose group)(BP is Biological Process; CC is Cellular Component; MF is Molecular Function).

For GO pathway analysis, the functions of significantly differentially expressed genes were annotated in this study using ClusterProfiler software (v 3.4.4), with *p* < 0.05 as the threshold for significant enrichment. One hundred and sixty four GO terms were significantly enriched in the selected DEGs in the ME and LE groups, of which those related to production performance included steroid metabolic process, phospholipid homeostasis, extracellular matrix, iron ion binding, etc. The selected DEGs in the HE and LE groups were significantly enriched to 284 GO items, including extracellular matrix organization, skeletal system development, growth factor activity, extracellular matrix structural constituent, etc. The selected DEGs in the HE and ME groups were significantly enriched to 238 GO items, including lipoprotein metabolic process, immune response, cell–cell signaling, and so on. The top 10 terms with the highest significance (*p* < 0.05) in each functional classification are shown (Figures 9A–C).

### 3.11 Joint analysis of the metagenome and transcriptome

**Figure 10:**
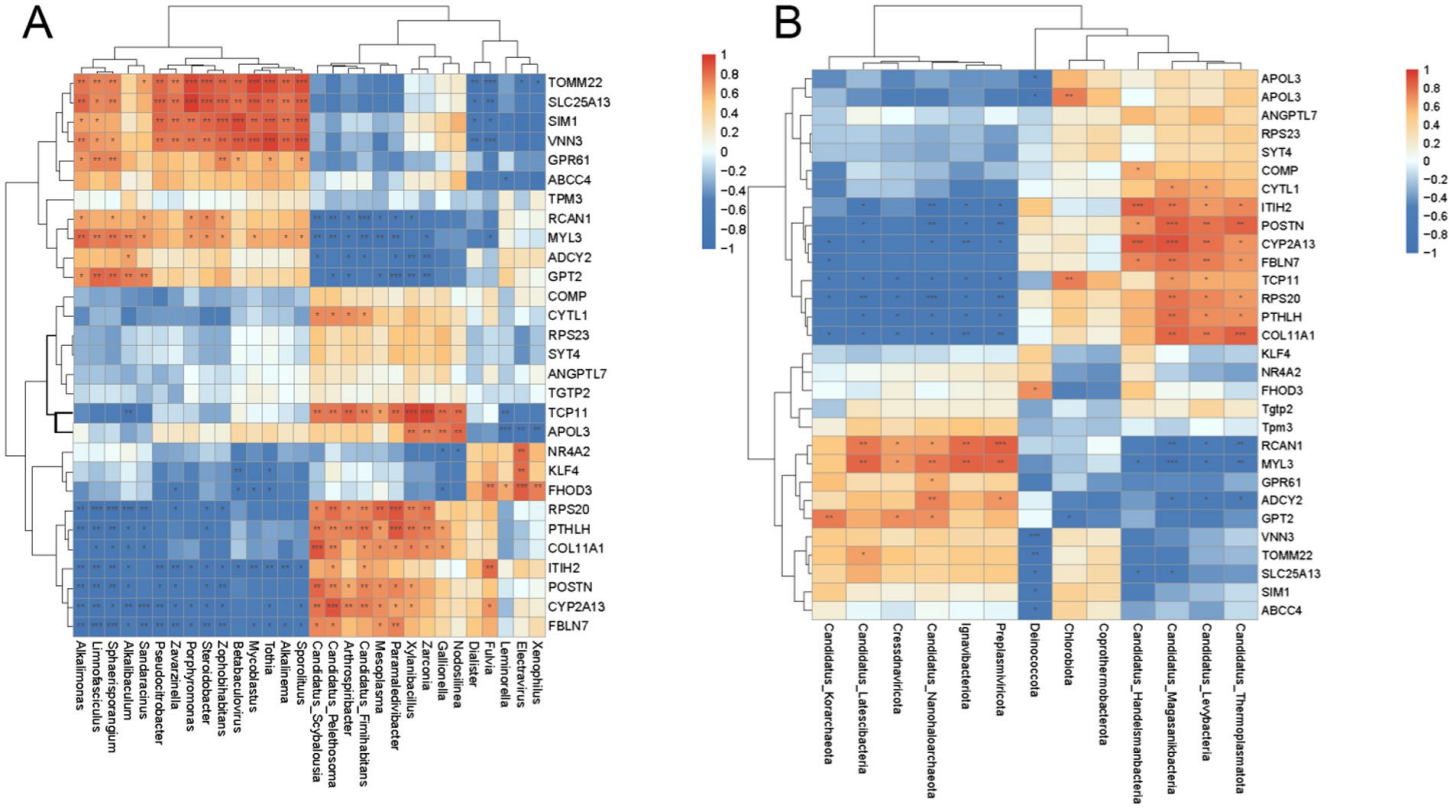
The top 30 items with significant differences in metagenomics and transcriptome analysis were: **(A)** genus level **(B)** gate level. The horizontal and vertical axes are the top differences after screening. “*” means correlation *p*-value < 0.05, “**” means correlation *p*-value < 0.01, “***” means correlation *p*-value < 0.001.

To visualize the similarities and differences in the correlation of the two omics elements, the two omics elements were analyzed by Spearman correlation hierarchical clustering analysis and presented in the form of a correlation hierarchical clustering heatmap. The top 30 entries of significant differences between metagenomics and transcriptomes were selected separately, and their correlations were calculated and plotted as significant correlation hierarchical clustering heatmaps. Figure 10 shows that at the phylum and genus level some genes related to growth performance are significantly correlated with microorganisms, such as COL11A1 (Collagen alpha-1 (XI) chain), POSTN (Periostin), and PTHLH (Osteostatin). As shown in Figure 10A at the genus level Candidatus_Scybalousia showed a highly significant positive correlation with the COL11A1 gene (*p* < 0.001), Candidatus_Pelethosoma showed a significant positive correlation with the COL11A1 gene (*p* < 0.01) and Candidatus_ Fimihabitans, Mesoplasma, Paramaledivibacter, Xylanibacillus, Zarconia, Gallionella were positively correlated with COL11A1 (*p* < 0.05). Candidatus_Scybalousia, Candidatus_Pelethosoma, and Candidatus_Fimihabitans were significantly and positively correlated (*p* < 0.01) with the POSTN gene, Arthrospiribacter, Mesoplasma, Paramaledivibacter, Xylanibacillus were positively associated with POSTN (*p* < 0.05). While PTHLH showed a highly significant positive correlation with Paramaledivibacter (*p* < 0.001), with Zarconia, Xylanibacillus, Candidatus_Scybalousia, Candidatus_Pelethosoma, Arthrospiribacter, Candidatus_Fimihabitans were significantly positively correlated (*p* < 0.01) with Mesoplasma, Gallionella (*p* < 0.05).

At the Phylum level (Figure 10B) COL11A1 was highly significantly positively correlated with Candidatus_Thermoplasmatota (*p* < 0.001) and significantly positively correlated with Candidatus_Magasanikbacteria, Candidatus_Levybacteria (*p* < 0.01) POSTN showed highly significant positive correlation (*p* < 0.001) with Candidatus_Magasanikbacteria, significant positive correlation (*p* < 0.01) with Candidatus_Levybacteria, Candidatus_Thermoplasmatota, and significant positive correlation (*p* < 0.01) with Candidatus _Handelsmanbacteria was positively correlated (*p* < 0.05). PTHLH was significantly positively correlated (*p* < 0.01) with Candidatus_Magasanikbacteria, and positively correlated (*p* < 0.05) with Candidatus_Levybacteria, Candidatus_ Thermoplasmatota were positively correlated (*p* < 0.05).

## 4 Discussion

The Qinghai yak is a unique species living in the Qinghai-Tibetan Plateau region at an altitude of 2,500–6,000 m above sea level (Ma J. et al., 2020). The region is characterized by very harsh climatic conditions, with large temperature fluctuations and severe hypoxia. In addition, on the plateau, its main diet consists of herbaceous plants, such as barley grass in the alpine grasslands and highland dwarf tarragon grass. These plants usually contain high fiber and hard shells, in which cellulose and hemicellulose are difficult to fully digest and absorbed by yaks. This climatic condition as well as food affects their growth, development, and nutrient metabolism (Shaoke et al., 2024). Therefore, the growth performance of yaks cannot be maximized under natural grazing conditions, which makes the yak industry stagnant. Therefore, there is an urgent need to change the feeding pattern to promote the growth of yaks and thus the development of the livestock industry in the Tibetan Plateau. According to previous studies, feeding enzymes can improve the growth performance of livestock and poultry (Xue et al., 2024). The study showed that with the concentration of enzymes, the growth performance of livestock and poultry can be improved. The study showed that as the concentration of enzyme increased, the growth performance also increased, and the HE group had the fastest monthly growth rate and monthly weight gain compared with the LE and ME groups. This suggests that appropriately increasing the concentration of compound enzyme preparation in the feed under captive conditions can improve the growth performance of yaks so that the excellent traits of yaks can be utilized and the economic benefits of herders can be improved.

Dynamic changes in serum biochemical indicators can reflect the level of digestion and absorption of nutrients, health status and growth performance of livestock and poultry, as well as changes in the function of some tissues and organs (Kokore et al., 2021). Immunoglobulins in serum are mainly responsible for the immune response to antigens (Ballow, 2010). In this experiment, globulin increased with the increase of enzyme concentration, which indicates that the moderate increase of enzyme dose can improve the immune ability. The transaminase index of HE group was lower than ME and LE group, and the role of transaminase in the liver is to catalyze amino acid deamination, and the increase of transaminase activity indicates the decrease in the ability to utilize the proteins, which indicates that the increase of enzyme dose enhances the immunity of the tissue organs. This suggests that the addition of enzyme dose enhances the ability to utilize proteins (Suciu et al., 2020). AST and alkaline phosphatase (ALP) are widely distributed in animals and are found primarily in the myocardium and to a lesser extent in the liver; AST is an important indicator of liver function, and high levels of ALT may indicate abnormalities in liver function, whereas ALP activity is primarily indicative of myocardial function; therefore, changes in serum aminotransferase concentrations are often used as a basis for determining normal liver and heart function (Detrich et al., 2010). In the present experiment, the effect of the enzyme complex on the serum transaminase level of yaks showed a decreasing trend but was not significant, which indicated that the enzyme complex did not adversely affect the heart and liver functions of yaks. The content of TP in serum can reflect the protein metabolism of the liver and the protein deposition status of the organism, and there is a correlation between it and the immunoglobulin content. Some experiments have shown that the addition of complex enzyme preparation can increase the concentration of total protein and albumin (Long et al., 2021). This is consistent with the results of this experiment Gao Yuhong et al. Reported that the addition of enzyme complexes to diets increased blood glucose levels by 5.9–35.5% (Gao Yuhong et al., 2000). However, blood glucose levels did not increase in this experiment, which may be due to the fact that as the dose of the enzyme preparation increases it affects the rate of digestion and absorption of food in the gastrointestinal tract, resulting in a slower release of glucose into the bloodstream, which affects the fluctuation of blood glucose levels. In addition, rumen pH has an important effect on yak health and production performance, in this experiment about rumen fluid pH measured results were HE group > ME group > LE group, and the rumen fluid pH of HE group was significantly higher than that of LE group (*p* < 0.05), which was consistent with the experimental results of Yanling Li, however, the experimental results of Liansheng Zhao, Jing Lin, Xiaojiao Che, et al. Demonstrated that the addition of enzyme preparation decreased rumen pH (Yanling et al., 2015; Jing et al., 2017; Liansheng et al., 2018; Xiaojiao et al., 2022). The reason for this result in this experiment may be that the addition of enzyme doses allowed the complex carbohydrates in the feed to be broken down into simple sugars more rapidly, thus reducing the accumulation of organic acids (e.g., lactic acid) (Wu et al., 2024). Accumulation of lactic acid is one of the main causes of lower rumen pH. By reducing lactic acid production, enzymes help to maintain a stable or rising rumen pH.

Muscle pH, meat color, drip loss and cooking loss are all key factors in assessing meat quality. pH levels can indicate the rate of muscle glycogenolysis after butchering, which affects meat color, tenderness and overall palatability and is a key determinant of meat quality (Redoy et al., 2020). It has been reported that there is a significant correlation between meat color and meat shelf life, with the better the color, the longer the shelf life (Yinzhu et al., 2021; Yonggang et al., 2021). Drip loss and cooking loss also reflect the tenderness of muscle, are important indicators of muscle water absorption, and are negatively correlated with meat quality (Changxi et al., 2021). Studies have shown that supplementation of exogenous enzymes in ruminant diets can improve meat tenderness and color to varying degrees (Elbedawy et al., 2011; Haocheng et al., 2021). In this experiment although none of the meat color indexes were significantly different, there was a tendency to increase meat color L, a and b at 24H, and the better the muscle quality, the brighter the muscle color, indicating that the flesh color could be improved with the increase of enzyme dosage in the diet. Meanwhile, the HE group was higher than the ME and LE groups in terms of drip loss rate 48 h and cooking loss rate indexes, which reflected that a moderate increase in enzyme dosage could improve muscle tethering force. Moreover, the carcass weight of HE group was higher than that of ME and LE groups probably due to the accelerated growth rate of yaks. In addition, muscle pH, shear force, and eye muscle area did not change much with increasing enzyme dose.

Genomics enables the study of compositional and functional characteristics of rumen microorganisms (Shinkai et al., 2024). By annotating the genes encoding carbohydrases, we can quickly and comprehensively understand the effects of adding different enzyme doses on the rumen microbiota of yaks. In this experiment, the dominant microbial composition of the three groups at the phylum level was Bacteroidota, Bacillota, Kiritimatiellota, and Pseudomonadota, among others. They have high potential for cell wall degradation and play a key role in carbohydrate degradation and metabolism, thus promoting animal growth and development (Pisaniello et al., 2023; Macdonald et al., 2024). Moreover, the HE and ME groups had higher numbers of two microorganisms, Bacteroidota and Bacillota, than the LE group, which suggests that increasing the dose of enzymes accelerated the degradation of carbohydrates and fibers, and accelerated the nutrient uptake. At the genus level, the dominant microbial genera in the three treatment groups were Prevotella, Methanobrevibacter, Oscillibacter, and Fibrobacter. Prevotella plays an important role in carbohydrate and nitrogen metabolism in the rumen, which may contribute to methane mitigation strategies (Kim et al., 2017); Methanobrevibacter is a producer of methane and is essential for gas homeostasis in the rumen (Savant and Ranade, 2004); Oscillibacter may have a role in low methane emission groups (Aguilar et al., 2020); whereas Fibrobacter plays an important role in wood fiber degradation (Neumann and Suen, 2018). In addition the number of Prevotella was lower in the LE group than in the ME and HE groups, which indirectly suggests that the increase in enzyme dosage improved carbohydrate and nitrogen metabolism. Moreover, this is also consistent with the results of the α-diversity analysis, where the rumen microbial community had the highest ACE index and Chao1 in the HE group but the lowest in Simpson and Shannon, which may be caused because the enzyme preparations led to the overgrowth or competitive advantage of certain microbial groups, as in the case of dominant microbes at the phylum level and genus level, which in turn led to the reduction or suppression.

Metagenomics can be used to annotate genes encoding CAZymes (Dong and Strous, 2019). Therefore, in this study, the genes encoding CAZymes in rumen microbes were annotated and found to be the most abundant in GHs, GTs, and CEs in the three treatment groups. Carbohydrates in ruminant diets provide fermentation substrates for rumen microorganisms, and in the presence of rumen microorganisms, carbohydrates make up a large portion of the diet, usually reaching 60% or even higher (Xuezhao et al., 2022). The complex carbohydrates are broken down into monosaccharides and oligosaccharides catalyzed by various hydrolytic enzymes, and these oligosaccharides are then rapidly broken down by microorganisms into pyruvic acid, which is fermented through metabolic pathways to ultimately produce acetic acid and propionic acid (Monika et al., 2022). Among them, GHs play an important role for the utilization of carbohydrates as they hydrolyze glycosidic bonds, thus helping the organism to obtain the required nutrients and energy (van Wyk et al., 2017). GTs are involved in the synthesis of glycoproteins, glycosaminoglycans, etc. Which play an important role in cellular structure and function and are essential for growth, development and metabolic regulation (Li Y. et al., 2019). CEs are able to hydrolyze carbohydrate ester bonds, such as playing a role in acetylation modification of plant cell walls. They may also facilitate the disruption of glycosidic bonds of polysaccharides by GHs and PLs by altering the structure and properties of plant cell walls (Kumar and Wensheng, 2018). PLs help accelerate the degradation and utilization of polysaccharides (Chen et al., 2023). CBMs do not have catalytic activity *per se*, but they are able to bind and localize on polysaccharide molecules to enhance the degradation efficiency of the degrading enzymes. Investigation of the CAZymes family showed that there were differences between the three treatment groups as the dose level of the complex enzyme preparation increased. The HE group had more GH families, including glucosidases (GH5, GH33, GH30_8, GH43_16, GH30_3) that act on glycosidic bonds of various substrates, and amylases (GH20, GH13_36, GH13_20, GH13_39) that act mainly on hydrolyzing polysaccharides and oligosaccharides, in addition to the GH families that help the enzyme with its substrate CBM9 and CBM35, which bind, and GT94, which catalyzes the glycosyl transfer reaction, were more abundant than in the ME and LE groups. Moreover, the 12 GH families were also significantly higher in the ME group than in the LE group, suggesting that yaks in the high-dose enzyme group may have a greater ability to catabolize cellulose and hemicellulose because glucosidase hydrolyzes not only lactose but also plant polysaccharides catalyzed by microbial cellulases (Lee et al., 2012). Previous studies have shown that glucosidases, especially the GH5 family, contain a variety of cellulases that can effectively degrade cellulose. The enzymes of this family usually have a cellulose binding domain, which allows them to better bind to cellulose, thereby improving the binding ability of the enzyme to the substrate (Ma L. et al., 2020). In addition, some studies have shown that CAZymes can regulate the use of carbohydrates and affect the energy acquisition and utilization of muscle cells (Vincent et al., 2014). Therefore, we speculate that the difference in CAZymes may affect the synthesis and decomposition of saccharide, thereby affecting the energy supply and metabolic processes of muscle cells.

Through transcriptomics studies, it is possible to reveal the expression levels and regulation of genes under different doses of enzyme preparations, and to understand the extent of the effects of varying enzyme preparations on genes related to the regulation of muscle growth, to understand better the mechanism of the regulation of its growth performance (Macken et al., 2021). In this experiment, it was found that many of the DEGs have been found to have important roles in regulating the growth and development of muscle tissues, including COL11A1, FOXO1, POSTN, PTHLH, and other genes related to the regulation of the development of the muscle, bone, and nervous system. These genes were up-regulated in comparing the high-dose enzyme preparation group and the low-dose enzyme preparation group. The DEGs were also analyzed by GO and KEGG enrichment and found that the HE and LE groups were significantly up-regulated in the Protein digestion and absorption, ECM-receptor interaction, and PI3K-Akt signaling pathway pathways. Among them, Protein digestion and absorption are closely related to growth promotion, and feeding *Bacillus subtilis* to improve the growth performance of pigs in Li′s experiment revealed that the Protein digestion and absorption pathway was significantly enriched, which was consistent with the results of this experiment (Li et al., 2023). Bao Y et al. found that the ECM-receptor interaction pathway is involved in regulating biological processes such as cell adhesion, migration, and proliferation by modulating the interaction between cells and extracellular matrix (Bao et al., 2019). These processes are essential for cell proliferation and growth. Elia also indicated that by acting on skeletal muscle fibers, the GH/IGF growth axis further activates the PI3K-Akt signaling pathway signaling pathway, promotes protein synthesis, and increases myofiber size (Elia et al., 2007). In the HE and ME groups, the PI3K-Akt signaling pathway was further activated. In contrast, the PI3K-Akt signaling pathway, Insulin secretion, and ECM-receptor interaction pathways were significantly up-regulated in the HE versus ME group. The insulin secretion pathway is an important biological process that regulates insulin secretion, which can affect growth performance by regulating blood glucose levels and promoting cellular uptake of glucose (Kalwat and Cobb, 2017). This is indirectly evidenced by the fact that blood glucose concentrations were significantly lower in the HE than in the LE and ME groups in the text. Finally DEGs from ME and LE groups were significantly enriched in the PPAR signaling pathway, ECM-receptor interaction, and Thyroid hormone synthesis, and the PPAR signaling pathway is a key signaling pathway in regulating IMF deposition in Cao et al. experiment that PPAR signaling pathway has been demonstrated to be a key signaling pathway regulating IMF deposition (Cao et al., 2023). The thyroid hormone synthesis pathway plays an important role in the growth performance of livestock and poultry, and the addition of thyroxine to the feed of chickens affected body weight gain and feed utilization efficiency by May et al.(May, 1980). From the above results, it was found that increasing the enzyme dose significantly up-regulated the expression levels of growth-related genes such as COL11A1, FOXO1, POSTN, and PTHLH, which in turn enhanced the ECM-receptor interaction, PI3K-Akt signaling pathway, PPAR signaling pathway, and other growth-related pathway expression, promoting animal growth and development. This is consistent with the results of phenotypic shape data and argues the effect of different doses of enzyme preparation on growth performance at the gene level.

In order to investigate the relationship between the transcript levels of genes in the longest dorsal muscle of yaks fed at dietary protein levels and their microorganisms, the present study was conducted to correlate the expression data of the two. By integrating the transcriptomic and macrogenomic information, we can deepen the understanding of the mechanism of dietary enzyme supplementation affecting the growth performance of yaks, and provide a deeper theoretical reference for the genetic improvement and fattening of yaks in the future. The results of the analysis revealed that there were significant correlations between the genes COL11A1, POSTN, and PTHLH at the phylum and genus levels in a variety of microbiota, and that the COL11A1 gene encodes the α1 chain of type XI collagen, which is a kind of collagen that is mainly found in cartilage and other connective tissues. This collagen plays an important role in maintaining tissue structure and providing support and elasticity. COL11A1 gene expression is regulated by a variety of growth factors, such as transforming growth factor β (TGF-β) (Niloufar et al., 2024). In addition COL11A1 plays an important role in bone development. It is not only involved in the assembly of collagen fibers, but also affects the morphology and strength of bone (Nallanthighal et al., 2021). Therefore, the expression level of COL11A1 directly affects the formation and growth of bone tissue and plays a key role in cell proliferation, differentiation, and growth. Periostin (POSTN) is an extracellular matrix (ECM) protein belonging to the tumor growth factor (TGF) family, which is mainly secreted by osteoblasts. During skeletal development, Periostin expression increases significantly, especially in the periosteum and bone tissue. J et al. Found that Periostin, by regulating collagen cross-linking and fiber formation, is able to enhance the stability and function of the bone matrix by interacting with bone morphogenetic proteins (BMPs), thus supporting bone tissue development and repair (Conway et al., 2014). It has also been shown that Periostin is not only expressed in osteoblasts but is also involved in the proliferation process of other cell types. For example, in lung fibroblasts, the addition of recombinant Periostin protein promotes cell proliferation (Yoshihara et al., 2020). This enhancement of cell proliferation may be related to a series of signaling pathways activated by Periostin through integrin receptors such as αvβ3 and α6β4, which include the PI3K-AKT and FAK signaling pathways, which explains why the PI3K-Akt pathway was up-regulated in the high-dose group (Adrian et al., 2022). Activation of these signaling pathways promotes cell growth and migration, which in turn affects growth performance. The PTHLH gene encodes a hormone known as PTHrP (parathyroid hormone-related protein). PTHLH has a particularly prominent role in bone development. Studies have shown that it promotes the development of osteoblasts (bone-producing cells) and chondrocytes, which affects the rate and quality of bone growth. For example, in mouse models, the deletion of the PTHLH gene leads to immediate death after birth, mainly due to insufficient thoracic development, suggesting the importance of this gene in bone development (Martin et al., 2021; Scheffer-Rath et al., 2023). In addition PTHLH gene expression is regulated by a variety of internal and external factors. Transforming growth factor β1 (TGF-β1) has been found to increase the protein expression of PTHLH, which further promotes the proliferation and differentiation of related cells (He et al., 2018). This regulatory mechanism suggests that PTHLH is not only a structurally and functionally relevant hormone, but may also function as a signaling mediator of growth factors in cell–cell interactions. Moreover, all three genes, COL11A1, POSTN, and PTHLH, were up-regulated in the high-dose complex enzyme preparation group. Therefore, we hypothesized that microorganisms in yak could influence host growth and development by regulating the expression of these genes.

## 5 Conclusion

In this study, we compared the effects of the levels of compound enzyme preparations in the ratio on the production performance and serum biochemical indexes of yaks and explored their effects on the microbial community composition and gene expression by metagenomics and transcriptomics. The results showed that the average daily weight gain of yaks was significantly increased by the high dose of compound enzyme preparation in the ratio compared with the low dose, the rumen fluid pH of the HE group was significantly higher than that of the LE group, and in terms of serum biochemical indexes, the calcium and glucose concentrations of the LE and ME groups were significantly higher than that of the HE group. However, in terms of meat quality, the differences among the three groups were not significant for each index. At the taxonomic level of phylum and genus, the level of complex enzyme preparation had no significant effect on the dominant microorganisms. However, analysis by the CAZymes family showed significant differences in GHs, CTs, and CEs among the three groups. Differentially expressed mRNAs were significantly enriched in signaling pathways such as ECM, PI3K-Akt, PPAR, and Protein digestion and absorption by GO and KEGG pathway analysis. Finally, based on the combined metagenomics and transcriptomics analysis, it was understood that some of the microorganisms were significantly associated with growth and metabolism-related genes such as COL11A1, POSTN, and PTHLH. In conclusion, this study reveals how compound enzyme preparation affects yak performance and meat quality by changing the structure and function of rumen microbial community. This provides a scientific basis for optimizing feeding management. It also provides in-depth data on how the microbial community and its metabolites affect host physiology through a combination of metagenomics and transcriptomics. These data help to construct a microbe-host interaction model for ruminant nutrition. Future research will focus on dose optimization, long-term impact assessment and in-depth discussion of microbial functions to further promote scientific progress in this field.

## Data Availability

All the raw datas were submitted to the NCBI Sequence Read Archive (SRA) database (Accession number: PRJNA1156913).
