# Supplementary material for: The effects of different doses of compound enzyme preparations on the production performance, meat quality and rumen microorganisms of yak were studied by metagenomics and transcriptomics
Source: Front Microbiol. 2024 Dec 11;15:1491551. doi: 10.3389/fmicb.2024.1491551 (PMC11670318; doi:10.3389/fmicb.2024.1491551)
Supplement: Supplementary file 1 [file Supplementary_file_1.docx]

**Supplementary Material**

# **1.Supplementary Material S1**

Supplementary Material S1 Composition and content of supplementary diet

| **Raw material name** | **LE** | **ME** | **HE** |
| --- | --- | --- | --- |
|  | **content%** | | |
| Corn ( Level 1 ) | 58.00 | 58.00 | 58.00 |
| 46 cottonseed meal | 11.80 | 11.80 | 11.80 |
| corn bran shotcrete | 3.00 | 3.00 | 3.00 |
| white lees | 3.00 | 3.00 | 3.00 |
| DDGS | 5.10 | 5.10 | 5.10 |
| Tong bran | 1.80 | 1.80 | 1.80 |
| soybean hull | 2.00 | 2.00 | 2.00 |
| tomato skin | 2.00 | 2.00 | 2.00 |
| 43 soybean meal | 4.30 | 4.30 | 4.30 |
| magnesium oxide | 0.20 | 0.20 | 0.20 |
| molasses | 3.00 | 3.00 | 3.00 |
| fine ground limestone | 1.50 | 1.50 | 1.50 |
| sodium chloride | 1.20 | 1.20 | 1.20 |
| calcium hydrogen phosphate | 0.80 | 0.80 | 0.80 |
| Expanded urea | 0.70 | 0.70 | 0.70 |
| sodium bicarbonate | 0.60 | 0.60 | 0.60 |
| Compound enzyme | 0.05 | 0.10 | 0.20 |
| Ruminate 1 % premix | 1.00 | 1.00 | 1.00 |

**2.Supplementary Material S2**

Supplementary Material S2: Comparison of body weight and monthly weight change among three treatment groups

| Item | Treatment group (unit: kg, Mean±SEM） | | | *P*-Value |
| --- | --- | --- | --- | --- |
|  | LE | ME | HE |  |
| Month 0 | 289.43±6.88^a^ | 286.5±5.01^a^ | 288.6±4.77^a^ | 0.658 |
| Month 1 | 305.71±8.77^a^ | 309.67±9.69^a^ | 314.8±4.76^a^ | 0.204 |
| Month 2 | 327.57±14.05^b^ | 324.17±7.68^ab^ | 339.8±10.5^a^ | 0.090 |
| Month 3 | 349.43±17.15^b^ | 352.67±10.6^b^ | 373.2±12.6^a^ | 0.027 |
| ∆ Month 1 | 16.29±7.65^b^ | 23.17±7.39^ab^ | 26.2±4.44^a^ | 0.610 |
| ∆ Month 2 | 21.86±9.58^a^ | 14.5±5.36^a^ | 25±8.4^a^ | 0.113 |
| ∆ Month 3 | 21.86±7.67^b^ | 28.5±9.48^ab^ | 33.4±2.41^a^ | 0.510 |
| Average daily gain | 0.631±0.182^b^ | 0.697±0.105^b^ | 0.891±0.138^a^ | 0.027 |

**3.Supplementary Material S3**

Supplementary Material S3: Statistical analysis of metagenomic sequencing data of each sample

| Treatment group | Sample name | Raw reads | Clean reads | Contigs | N50 (bp) | ORFs |
| --- | --- | --- | --- | --- | --- | --- |
| LE | LE 1 | 72,754,802 | 72,631,200.00 | 1,480,353.00 | 758.00 | 1,643,979.00 |
|  | LE 2 | 73,545,436 | 73,420,856.00 | 1,550,483.00 | 752.00 | 1,743,365.00 |
|  | LE 3 | 68,025,496 | 67,906,796.00 | 1,149,067.00 | 844.00 | 1,287,349.00 |
|  | LE 4 | 82,034,238 | 81,954,278.00 | 1,776,191.00 | 771.00 | 1,930,436.00 |
| ME | ME 1 | 79,586,832 | 78,751,738.00 | 1,548,964.00 | 818.00 | 1,776,504.00 |
|  | ME 2 | 73,300,888 | 73,174,116.00 | 1,500,128.00 | 765.00 | 1,657,596.00 |
|  | ME 3 | 70,297,996 | 70,183,984.00 | 1,407,285.00 | 784.00 | 1,606,111.00 |
|  | ME 4 | 75,322,506 | 75,197,764.00 | 1,014,394.00 | 919.00 | 1,230,828.00 |
| HE | HE 1 | 78,879,196 | 78,750,774.00 | 1,654,204.00 | 755.00 | 1,876,408.00 |
|  | HE 2 | 81,506,876 | 81,427,516.00 | 1,594,738.00 | 872.00 | 1,859,754.00 |
|  | HE 3 | 74,621,164 | 74,549,952.00 | 1,279,173.00 | 929.00 | 1,527,871.00 |
|  | HE 4 | 81,709,758 | 81,631,018.00 | 1,815,235.00 | 740.00 | 2,022,569.00 |
| Total | | 911,585,188 | 909,579,992.00 | 17,770,215.00 | 9,707.00 | 20,162,770.00 |
| Mean | | 75,965,432 | 75,798,332.67 | 1,480,851.25 | 808.92 | 1,680,230.83 |
| SD | | 4,698,036 | 4,664,460.76 | 238,493.08 | 66.95 | 243,202.01 |

**4.Supplementary Material S4**

Supplementary Material S4: Microbial composition of all samples at domain taxonomic level


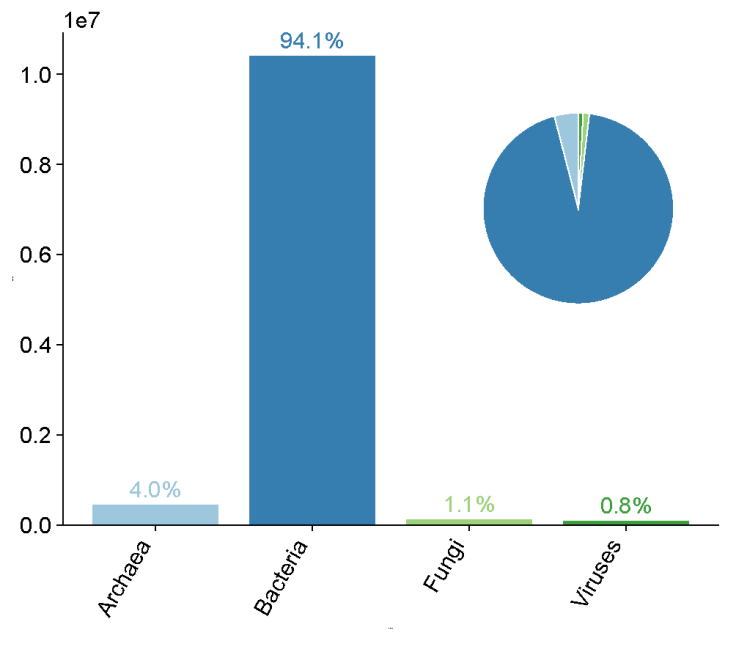


**5.Supplementary Material S5**

Supplementary Material S5: Statistical analysis of metagenomic sequencing data of each sample

| Treatment group | Sample name | Raw reads(M) | Clean reads(M) | Clean Base(G) | Q30 (%) | GC Content |
| --- | --- | --- | --- | --- | --- | --- |
| LE | LE 1 | 47.73 | 46.85 | 6.94 | 95.69 | 54.33 |
|  | LE 2 | 47.33 | 46.61 | 6.93 | 95.6 | 53.52 |
|  | LE 3 | 48.07 | 47.25 | 7.01 | 95.71 | 53.83 |
|  | LE 4 | 47.94 | 46.78 | 6.9 | 95.69 | 54.87 |
| ME | ME 1 | 48.35 | 47.33 | 7.01 | 93.4 | 53.35 |
|  | ME 2 | 48.61 | 47.57 | 7.04 | 93.42 | 54.53 |
|  | ME 3 | 47.24 | 46.45 | 6.9 | 93.37 | 53.96 |
|  | ME 4 | 48.39 | 47.46 | 7.03 | 93.53 | 54.34 |
| HE | HE 1 | 48.35 | 47.29 | 7 | 93.48 | 54.43 |
|  | HE 2 | 46.27 | 45.39 | 6.73 | 93.39 | 53.68 |
|  | HE 3 | 48.28 | 47.31 | 7.01 | 93.46 | 53.42 |
| Total | | 526.56 | 516.29 | 76.5 | 1036.74 | 594.26 |
| Mean | | 47.87 | 46.94 | 6.95 | 94.25 | 54.02 |
| SD | | 0.69 | 0.63 | 0.09 | 1.13 | 0.51 |

**6.Supplementary Material S6**

Supplementary Material S6: Statistical analysis of transcriptome sequencing data of each sample

| Treatment group | Sample name | Total Reads | Total Mapped | Multiple mapped | Unique Mapped | Positive_Map | Negative_Map |
| --- | --- | --- | --- | --- | --- | --- | --- |
| LE | LE 1 | 46846304 | 43858022(93.62%) | 7903653(16.87%) | 35954369(76.75%) | 17934923(38.28%) | 18019446(38.47%) |
|  | LE 2 | 46608830 | 43060183(92.39%) | 6881995(14.77%) | 36178188(77.62%) | 18056730(38.74%) | 18121458(38.88%) |
|  | LE 3 | 47253078 | 43849622(92.80%) | 7087159(15.00%) | 36762463(77.80%) | 18362723(38.86%) | 18399740(38.94%) |
|  | LE 4 | 46776078 | 43691604(93.41%) | 7354213(15.72%) | 36337391(77.68%) | 18127015(38.75%) | 18210376(38.93%) |
| ME | ME 1 | 47325878 | 43226767(91.34%) | 6728237(14.22%) | 36498530(77.12%) | 18214777(38.49%) | 18283753(38.63%) |
|  | ME 2 | 47574442 | 44067160(92.63%) | 6979931(14.67%) | 37087229(77.96%) | 18512265(38.91%) | 18574964(39.04%) |
|  | ME 3 | 46446624 | 42862068(92.28%) | 7033503(15.14%) | 35828565(77.14%) | 17868810(38.47%) | 17959755(38.67%) |
|  | ME 4 | 47460970 | 44212646(93.16%) | 7059937(14.88%) | 37152709(78.28%) | 18536420(39.06%) | 18616289(39.22%) |
| HE | HE 1 | 47288752 | 43753819(92.52%) | 6631746(14.02%) | 37122073(78.50%) | 18519315(39.16%) | 18602758(39.34%) |
|  | HE 2 | 45393612 | 41946893(92.41%) | 5899789(13.00%) | 36047104(79.41%) | 17999119(39.65%) | 18047985(39.76%) |
|  | HE 3 | 47306736 | 43753593(92.49%) | 6259033(13.23%) | 37494560(79.26%) | 18713122(39.56%) | 18781438(39.70%) |
| Total | | 516281304 | 478282377 | 75819196 | 402463181 | 200845219 | 201617962 |
| Mean | | 46934664 | 43480216 | 6892654 | 36587562 | 18258656 | 18328906 |
| SD | | 629024 | 657897 | 530180 | 567027 | 284773 | 282490 |
